# Nutraceutical supplement slim reshaped colon histomorphology and reduces *Mucispirillum schaedleri* in obese mice

**DOI:** 10.3389/fmicb.2025.1494994

**Published:** 2025-04-01

**Authors:** Jessica Alves Freitas, Victor Nehmi Filho, Aline Boveto Santamarina, Gilson Masahiro Murata, Lucas Augusto Moyses Franco, Joyce Vanessa Fonseca, Roberta Cristina Martins, Gabriele Alves Souza, Gabriela Benicio, Isabella Mirandez Sabbag, Esther Alves de Souza, José Pinhata Otoch, Ana Flávia Marçal Pessoa

**Affiliations:** ^1^Laboratório de Produtos e Derivados Naturais, Laboratório de Investigação Médica-26 (LIM-26), Departamento de Cirurgia, Faculdade de Medicina da Universidade de São Paulo, São Paulo, SP, Brazil; ^2^Pesquisa e Desenvolvimento Efeom Nutrição S/A, São Paulo, SP, Brazil; ^3^Universidade de São Paulo Faculdade de Medicina da Universidade de São Paulo, Departamento de Clínica Médica, Laboratório de Nefrologia (LIM-29), São Paulo, SP, Brazil; ^4^Universidade de São Paulo Instituto de Medicina Tropical de São Paulo, Departamento de Doenças Infecciosas e Parasitárias, Laboratório de Parasitologia Médica (LIM-46), São Paulo, SP, Brazil; ^5^Universidade de São Paulo Instituto de Medicina Tropical de São Paulo, Departamento de Doenças Infecciosas e Parasitárias, Laboratório de Investigação Médica em Protozoologia, Bacteriologia e Resistência Antimicrobiana (LIM-49), São Paulo, SP, Brazil; ^6^Universidade de São Paulo Faculdade de Medicina da Universidade de São Paulo, Departamento de Patologia, Laboratório de Neurociência (LIM-01), São Paulo, SP, Brazil; ^7^Hospital Universitário da Universidade de São Paulo, Faculdade de Medicina da Universidade de São Paulo, São Paulo, SP, Brazil; ^8^Instituto Botânio, São Paulo, Brazil

**Keywords:** nutraceutical, prebiotic, beet pulp, coenzyme q10, berberine, obesity, gut microbiota

## Abstract

**Introduction:**

Bioactive compounds and whole foods have emerged as promising interventions to address gut microbiota dysbiosis linked to obesity. Compounds such as berberine and coenzyme Q10 are well-recognized for their roles in managing metabolic syndrome and exerting antioxidant effects, while beet pulp, rich in fiber and antioxidants, enhances gut health through additional prebiotic benefits.

**Methods:**

This study evaluated the effects of a nutraceutical supplement, Slim, on the modulation of gut microbiota in obese mice induced by a high-fat diet.

**Results:**

Our results demonstrated that Slim supplementation significantly improved lipid metabolism, reshaped colon histomorphology, and decreased levels of *Mucispirillum schaedleri*, which were correlated with VLDL-c and triglycerides.

**Discussion:**

We suggest these effects are driven by a duplibiotic effect, resulting from the synergistic action of the bioactive compounds.

## 1 Introduction

Supplements derived from natural compounds with therapeutic potential have garnered attention for their beneficial effects on various health conditions (Rahman et al., 2021; Guan et al., 2024). Obesity and overweight represent global public health challenges, being major risk factors for the development of metabolic syndrome, which includes disorders such as insulin resistance, high blood pressure, dyslipidemia, chronic low-grade inflammation, and the gastrointestinal tract histomorphology (Grundy, 2004; Kern et al., 2018; Leocádio et al., 2020; Freitas et al., 2024). Gut health, mediated by the integrity of the intestinal function and the balance of the microbiota, plays a crucial role in the regulation of immune system and energy metabolism, being essential for the maintenance of homeostasis in overweight or obese individuals (Abenavoli et al., 2019; Miron, 2019). The colon contains crucial structures that play an essential role in fostering a harmonious relationship between microbiota and its host. Lieberkühn crypts supporting cell regeneration and the secretion of enzymes, guarantee the functionality of the intestinal mucosa, crucial for the defense against pathogens (Ma et al., 2018). In parallel, Peyer’s patches monitor antigens and regulate the local immune response, preventing chronic inflammation that is commonly associated with obesity, thus promoting a balanced and healthy intestinal environment (Jung et al., 2010; Leocádio et al., 2020).

Gut microbiota has been widely studied due to its influence on host metabolism, particularly in obesity and overweight conditions (Esposito et al., 2003; Nehmi-Filho et al., 2023a). In obese individuals, the microbial composition is often altered, with a decrease in bacterial diversity and a predominance of microbial groups associated fat accumulation (John and Mullin, 2016; Saad et al., 2016). This can lead to increased intestinal permeability, favoring the translocation of bacterial endotoxins, such as lipopolysaccharide, which contribute to metabolic syndrome rises (Festi et al., 2014; Wang et al., 2024).

Bioactive compounds like FOS (Fructooligosaccharides), GOS (Galactooligosaccharides), yeast *β*-glucans, and silymarin in their formulation stand out, widely studied and recognized for their innumerous benefits from the gut microbiota and good consumers acceptance (Nehmi et al., 2021; Santamarina et al., 2022). Diversifying new nutrients and bioactive compounds is essential to maintaining and promoting biodiversity in the intestinal microbiota and thus preventing or improving diseases and symptoms related to obesity. Berberine (*Phellodendron amurense* Rupr., Rutaceae) stands out for its anti-inflammatory, hypoglycemic, and intestinal microbiota modulation properties, being especially useful in controlling type 2 diabetes and obesity (Habtemariam, 2020; Zhang et al., 2021). Coenzyme Q10 is an endogenous antioxidant compound with the role and its ability to improve mitochondrial function, contributing to the reduction of oxidative stress (Neergheen et al., 2017; Sifuentes-Franco et al., 2022). Beet or beetroot (*Beta vulgaris* L., Amaranthaceae) pulp is rich in insoluble fiber and bioactive compounds, such as nitrates, which can to improve intestinal health (Usmani et al., 2022; Adekolurejo et al., 2023). Combining these elements may result in a synergistic effect that helps prevent the emergence of metabolic diseases and alterations in the morphology and microbiota of the colon. This study aimed to evaluate the effects after 4 weeks of Slim supplementation on intestinal health and metabolic parameters, focusing on colon histomorphology and microbiota composition in obese mice induced by a high-fat diet.

## 2 Materials and methods

### 2.1 Supplement composition

The following elements constitute the supplement formulation (Patent: BR1020200161563) developed and tested in the present study: selenium (Se) 1.5%; FOS (Fructooligosaccharides) 30.10%; GOS (Galactooligosaccharides) 12.00% and 1,3/1,6- (β-glycosidic linkages) β-glucans from yeast (*Saccharomyces. cerevisiae*) 18% (Biorigin, Lençois Paulista, São Paulo, Brazil); chromium (Cr) 0.7%; beet pulp (*Beta vulgaris* L.) powder 30.10%; coenzyme Q10 1.5% and berberine (*Phellodendron amurense* Rupr.) extract 6.00%. Dietary reference values for the minerals described in this study were determined following guidance previously provided by the European Food Safety Authority (European Food Safety Authority, 2017). FOS (Sabater-Molina et al., 2009), GOS (Torres et al., 2010), yeast β-glucans (José Pereira, 2015; Andrade et al., 2016), beet pulp (ANVISA, 2018), coenzyme Q10 (Overvad et al., 1999; ANVISA, 2018) and berberine (Zamani et al., 2022) were determined based on previous studies considering the animal’s body area, determined by the equation: human equivalent dose (mg/kg) = animal does (mg/kg) × 12.33 (Jacob et al., 2022). Filtered mineral water in 2% carboxymethyl cellulose solution was used as an emulsifier for the formula components.

### 2.2 Ethics committee approval

This study was approved by the Research Ethics Committee of the University of São Paulo, São Paulo, Brazil, under approval numbers 1185/2018 and 1519/2020. All experiments were conducted in accordance with the guidelines of the National Institutes of Health.

### 2.3 Animal model and oral supplementation

**Figure 1:**
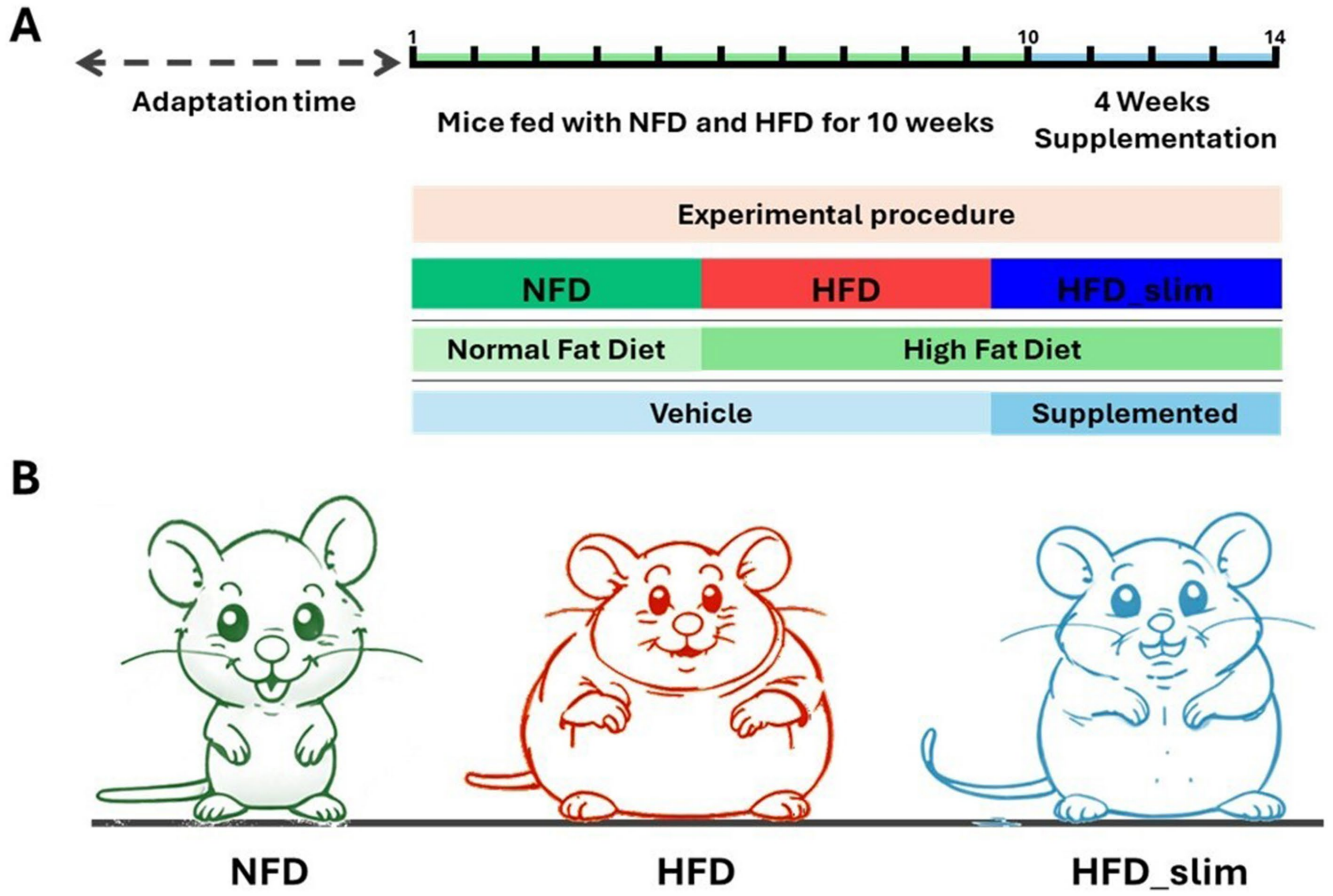
Schematic sketch of the experimental procedure and supplementation time. **(A)** Experimental schedule. **(B)** Experimental groups: NFD, Non-Fat Diet; HFD, High Fat Diet; HFD_Slim, High Fat Diet Slim.

Eight-week-old male C57BL/6 N mice were kept in an air-conditioned room at (24 ± 2) °C and subjected to a 12-h light/12-h dark cycle. These mice were subsequently separated into two groups: control and obese. The control group was fed an AIN-93 non-fat diet (NFD) of 4.1 kcal/g and was composed of 9% fat, 67% carbohydrate, and 15% protein. The High-Fat Group (HFD) consumed an AIN-93-based high-fat and high-sugar diet that contained 6.1 kcal/g and was composed of 25% fat, 49% carbohydrate, and 20% protein (Reeves et al., 1993; Prag Soluções *Biosciences*, Jaú, São Paulo, Brazil). Mice were fed these diets for 14 weeks and received chow and water *ad libitum.* In the 10th week, mice were divided into the following groups: NFD vehicle (*n* = 8), HFD vehicle (*n* = 7), and HFD_Slim supplemented (*n* = 8). NFD vehicle and HFD vehicle groups received 2% carboxymethyl cellulose (Vehicle) in mineral water. While HFD_Slim group received full supplementation. The groups were supplemented with 300 μL of the vehicle (NFD and HFD) and supplemented (HFD_Slim) diarily by gavage for 4 weeks (28 consecutive days). After 4 weeks of supplementation, mice were euthanized with an intraperitoneal injection of ketamine (100 mg/kg body weight) and xylazine (5 mg/kg body weight) (Figure 1).

### 2.4 Food intake, body mass, intraperitoneal glucose tolerance test (ipGTT), biochemistry parameters and cytokines

The food intake was measured during the 4 weeks supplementation. The groups body mass was measured weekly throughout the 14-week experimental period. We realizing the ipGTT, in the third week of supplementation after 6-h fasting period. Subsequently, each mouse received an intraperitoneal injection of a 10% glucose solution at a dose of 1.0 g/kg of body weight. Blood samples were then collected from the tail at time points 0, 30, 60, and 90 min, and blood glucose levels were measured using an Accu-Chek Active glucometer (Roche, Mannheim, Germany). Blood sample was collected during the euthanasia to serum analysis of cholesterol, triglycerides, aspartate aminotransferase (AST), alanine aminotransferase (ALT), gamma-glutamyl transferase (gamma-GT) and urea in the plasma were measured with a commercially available kit (Bioclin, Belo Horizonte, MG, Brazil). The low-density lipoprotein cholesterol (LDL-c) and very-low-density lipoprotein cholesterol (VLDL-c) levels were calculated according to Friedewald et al. (1972). The cytokines Interleucin-6 (IL-6) and Interleucin-10 (IL-10) levels in the colon of the mice were determined by enzyme-linked immunosorbent assays (ELISAs) according to the manufacturer’s instructions (R&D Systems, Inc., Shanghai, China).

### 2.5 Staining and histological analysis

The colon was removed, fixed in 4% formaldehyde for 24 h at room temperature, and processed as described by Pessoa et al. (2016). The 3-μm-thick sections of the colon were placed on glass slides and stained with Hematoxylin and Eosin (H&E) for histomorphology analysis. The slides were scanned using the Panoramic Scan scanner (3DHistech, Budapest, Hungary) analyzed using Slide Viewer software (3DHistech, Budapest, Hungary), and photographed at 200X and 400X magnifications.

### 2.6 Gut microbiota analysis

#### 2.6.1 Stool specimen collection and extraction

Stool samples were collected from the intestinal mucosa and stored in sterile tubes at −80°C until DNA extraction. Genetic material was acquired by DNA extraction. Approximately 250 mg of stool was sampled using the DNeasy PowerSoil kit (Qiagen, Hilden, Germany) following the manufacturer’s recommendations. The extracted DNA was stored at −20°C until sequencing library preparation.

#### 2.6.2 Sequencing library preparation

The fecal microbial component of mice was assessed based on partial 16S rDNA and 16S rRNA (V4 region) sequences amplified using a bacterial primer set 515F/806R for each DNA sample (Caporaso et al., 2011). DNA was quantified on the Qubit® 4.0 equipment using the dsDNA HS Assay kit (Thermo Fisher Scientific, Waltham, Massachusetts, USA). Microbiota characterization was performed by amplification of the bacterial 16S ribosomal V4 region. Using the Q5 High-Fidelity 2x Master Mix kit (New W England Biolabs, Ipswich, Massachusetts, USA), the primer sequences F515 (5′-CACGGTCGKCGGCGCCATT-3′), R806 (5′-GGACTACHVGGGTWTCTAAT-3′) and 50 ng of DNA, the bacterial DNA from the mice microbiota were amplified (Caporaso et al., 2011). Samples were purified with AMPure XP (Beckman Coulter, Brea, California, USA) and washed repeatedly with 70% ethanol. The success of the reaction was determined by the appearance of a positive band of 350 bp amplified on a 1.25% agarose gel. Library was quantified and created after measuring the concentration, using the Qubit® 4.0 equipment and the dsDNA HS Assay kit. The model was performed by the Ion Chef System (Thermo Fisher Scientific, Waltham, Massachusetts, USA), using the Ion 510™ & Ion 520™ & Ion 530™ Chef kit and library pool at 48pM. Sequencing was performed by an Ion S5 system, using the Ion S5 Sequencing kit and the Ion 520 Chip, according to the manufacturer’s instructions (Thermo Fisher Scientific, Waltham, Massachusetts, USA).

#### 2.6.3 16S rRNA gene data analysis

16S rRNA gene data, as well as diversity estimates, were processed and analyzed with Quantitative Insights Into Microbial Ecology (QIIME 2) software, version 2020.11 (Bolyen et al., 2019). Demultiplexed sequence data were filtered with DADA2 (using the q2-dada2 plugin) applying the default parameters: 200 bp length and an average Phred quality score ≥ 30, to generate amplicon sequence variants (ASVs) (Callahan et al., 2016). The phylogenetic tree was constructed by inserting the sequences into the Greengenes 13_8 reference tree, using the q2-fragment-insertion plugin, which employs the phylogenetic positioning insertion method enabled by SATé (SEPP) (Mirarab et al., 2011; Janssen et al., 2018). Alpha diversity metrics (Pielou evenness, Shannon, Simpson, and Chao1 indices, and Faith’s phylogenetic diversity) and beta diversity metrics (Jaccard distance, Bray–Curtis dissimilarity, Weighted_unifrac, and Unweighted_unifrac) were calculated using Q2-diversity after samples were rarefied to 27.745 sequences per sample (Faith, 1992). Principal Coordinate Analysis (PCoA) plot for each beta diversity metric was created using EM-peror (Vázquez-Baeza et al., 2013). ASVs were taxonomically assigned using the Q2 feature classifier (Bokulich et al., 2018) naive Bayesian classifier was applied against Greengenes 13_8 reference sequences with 99% OTUs (Operational Taxonomic Units) (Mandal et al., 2015).

### 2.7 Statistical analysis

Data were classified as parametric or nonparametric based on the Shapiro-Wilks test. When parametric, data were expressed as mean ± standard deviation (SD), when nonparametric, data were classified as median and interquartile range. For parametric data, comparisons were performed using the t-test with or without Welch’s correction. The Mann–Whitney test was applied when data were nonparametric. The NFD and HFD_Slim groups were compared with the HFD group. Comparisons between groups were performed using two-way analysis of variance (two-way ANOVA). Pearson correlation coefficients were calculated for the correlation between microbiota parameters and intestinal mucosa morphological structures. *R*^2^ values greater than 0.7 and less than −0.7 were considered strong correlations. The composition of the microbiological organization of the intestinal mucosa was briefly inferred through different levels of taxonomy, of which we can mention: phylum, genus, and species. For *α*-diversity, the correction of all *p*-values was performed by the Benjamini-Hochberg procedure. We compared groups (*β*-diversity) by permutation-based multivariate analysis of variance (PERMANOVA) using Bray–Curtis dissimilarity, Weighted_unifrac, Unweighted, and Jaccard and tested the homogeneity of variations between the composition of microbiota communities by PERMDISP (Permutational Analysis of Multivariate Dispersions). For all analyses, significance was determined as *p* < 0.05. Analyses were performed using STATA^®^ 14.0 (Stata Corp. LCC, College Station, TX, USA) and GraphPad Prism 9.0 (GraphPad Software, La Jolla, CA, USA) software.

## 3 Results

### 3.1 Slim nutraceutical effects on body mass, biochemistry parameters, and colon cytokine analysis after 4 weeks of supplementation

We observed a significant difference in body weight gain between the Normal Fat Diet (NFD) and High Fat Diet (HFD) groups during the 14 weeks of the experimental protocol (Supplementary Figures S1A,B). However, when we compared the groups at baseline (T0—week 10) and at the end of the supplementation period (T4—week 14), we did not find any differences (Table 1). While we present statistical differences in the data, we do not attribute the weight loss to the supplementation. This is because by the eighth week of the high-fat diet—before the supplementation began—statistical differences in weight between the groups were already observed (see Supplementary Figures S1A,B). The ipGTT and fasting glucose analyses showed statistically significant differences between the NFD group and the HFD groups. There was no statistically significant difference when comparing the HFD group with the Slim HFD group (Supplementary Figure S2A).

**Table 1:** Presentation of body mass data and biochemical parameters of Control and High Fat Diet groups after 4 weeks of supplementation.

Body mass in gram (g)			
	Time of supplementation		
	Before (T0)^a^	After (T4)^b^	*p* value
NFD	29.33 ± 0.98	30.56 ± 1.72	–
HFD	40.66 ± 3.70	42.54 ± 3.39	–
HFD_Slim	35.67 ± 2.87	35.71 ± 3.30	–

Biochemical data						
Parameters	NFD	HFD	HFD_Slim	NFD vs. HFD	NFD vs. HFD_Slim	HFD vs. HFD_Slim
	Mean ± SD	Mean ± SD	Mean ± SD	*p* value	*p* value	*p* value
Cholesterol (mg/dL)	137.90 ± 33.52	210.90 ± 16.52	196.30 ± 26.10	<0.001	<0.001	–
HDL-c (mg/dL)	61.00 ± 12.66	88.88 ± 6.01	77.38 ± 10.89	<0.001	<0.05	–
LDL-c (mg/dL)	54.13 ± 21.75	99.00 ± 13.44	98.13 ± 20.37	<0.001	<0.001	–
VLDL-c (mg/dL)	22.63 ± 1.76	22.88 ± 1.12	20.88 ± 1.24	–	0.050	<0.05
Triglycerides (mg/dL)	114.50 ± 8.31	113.00 ± 3.10	104.00 ± 5.75	–	<0.01	<0.05
Aspartate Transaminase – AST (U/L)	12.38 ± 5.73	12.38 ± 4.56	12.63 ± 3.54	–	–	–
Alanine Transaminase – ALT (U/L)	1.71 ± 1.38	11.88 ± 5.91	11.00 ± 30.38	<0.001	<0.001	–
Gamma-GT (U/L)	1.50 ± 1.06	2.37 ± 1.50	1.25 ± 0.88	–	–	–
Urea (mg/dL)	42.13 ± 7.69	38.50 ± 6.90	33.25 ± 7.70	–	–	–

^a^T0 = weight in gram (g) at week 10 before supplementation.

^b^T4 = weight in gram (g) at week 14 after supplementation.

The NFD group showed lower of cholesterol, HDL-cholesterol and LDL-cholesterol level when compared with the HFD and HFD_Slim. VLDL-cholesterol, triglycerides, AST, gamma-GT and urea levels did not differ between the NFD and HFD groups. We observed lower ALT level in NFD group when compared with HFD and HFD_Slim (Table 1).

We did not find significant differences between the HFD and HFD_Slim groups in the parameters of cholesterol, HDL-cholesterol, LDL-cholesterol, AST, ALT, gamma-GT and urea. We observed VLDL-cholesterol (*p* = 0.050) and triglycerides (*p* > 0.001) lower level in HFD_Slim group when compared with NFD and HFD (*p* < 0.05; *p* > 0.001, respectively) groups (Table 1).

We evaluated the levels of the colon cytokines IL-6 and IL-10 and calculated the IL-6/IL-10 ratio in the colon of the different groups after 4 weeks of supplementation. The levels of the IL-6 cytokine did not differ significantly between the groups. However, the IL-10 cytokine levels were significantly higher in the NFD group compared to the HFD and HFD_Slim groups. Additionally, the IL-6/IL-10 ratio was elevated in the HFD and HFD_Slim groups compared to the NFD group (see Supplementary Figures S2D–F).

### 3.2 Slim nutraceutical reshaped colon histomorphology in obese mice after 4 weeks of supplementation

**Figure 2:**
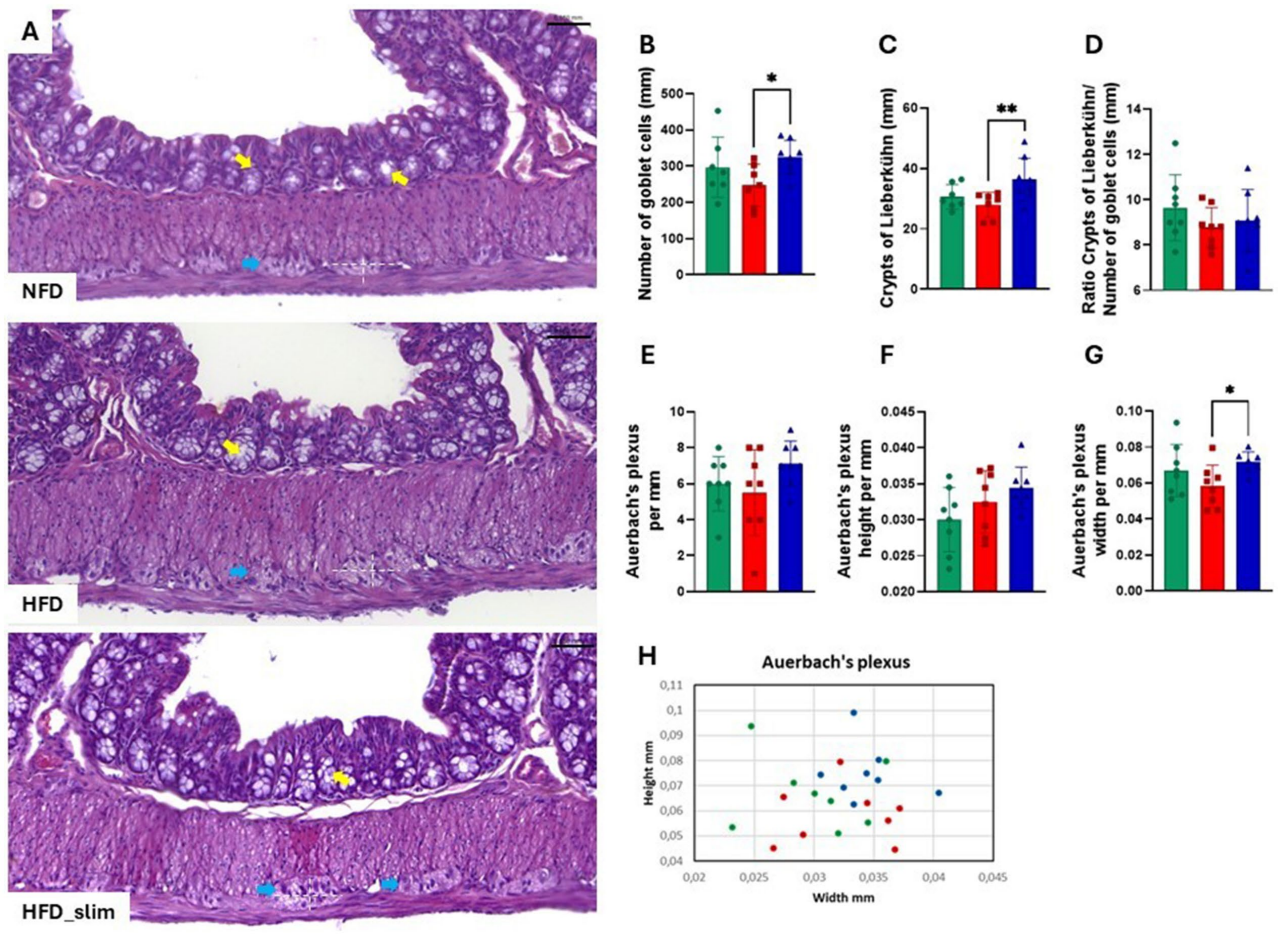
Assessment of histomorphological changes in the colon of control and high-fat diet groups after 4 weeks of supplementation. The H&E in stain shows **(A)** representative histological images of the colon. The graphics show **(B)** number of Goblet cells; **(C)** crypts of Lieberkühn; **(D)** ratio between the number of Goblet cells and crypts of Lieberkühn; **(E)** Auerbach plexus; **(F)** Auerbach plexus height **(G)** Auerbach plexus width **(H)** Scatter plot representing the Auerbach plexus. Scale bar = 0.050 mm, 200X magnifications. Statistical analyses were performed using the student’s t-test or Kruskal-Wallis test. Data are represented as mean ± SD. *n* = 7–8 animals per group. Differences were calculated about the HFD group. *p < 0.05; **p < 0.01. NFD, Non-Fat Diet; HFD, High Fat Diet; HFD_Slim, High Fat Diet Slim; mm, millimeters. Goblet cells, yellow arrows; Auerbach plexus, blue arrows; Dashed white, Auerbach plexus area.

**Figure 3:**
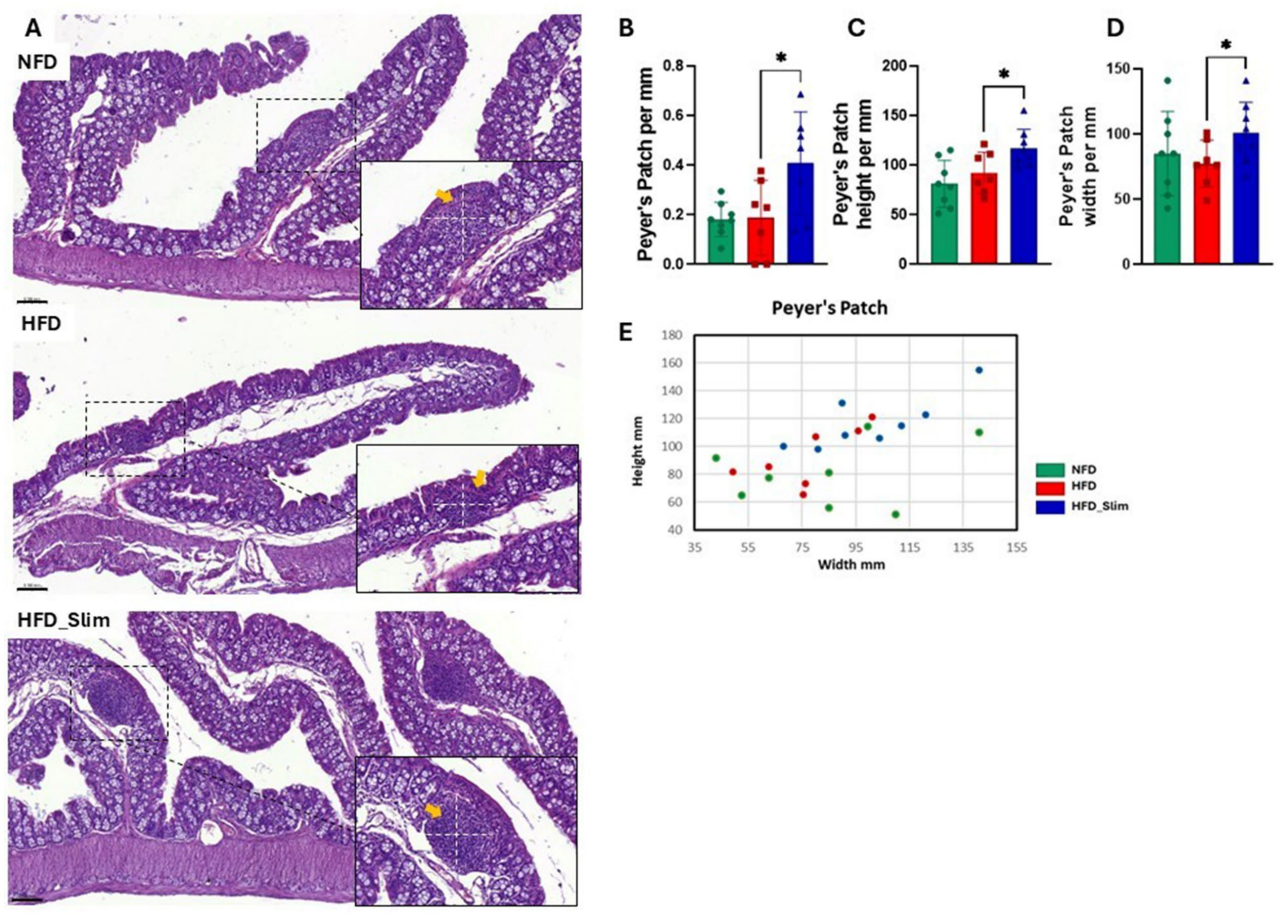
Peyer’s patches histomorphometry in the colon of Control and High-Fat Diet groups after 4 weeks of supplementation. The H&E in stain shows **(A)** representative histological images of the colon. The graphics show **(B)** Peyer’s patches; **(C)** Peyer’s patches height; **(D)** Peyer’s patches width; **(E)** Scatter plot representing Peyer’s patches. Scale bar = 0.050 mm, 200X and 400X magnifications. Statistical analyses were performed using the student’s t-test or Kruskal-Wallis test. Data are represented as mean ± SD. *n* = 7–8 animals per group. Differences were calculated about the HFD group. *p < 0.05; NFD, Non-Fat Diet; HFD, High Fat Diet; HFD_Slim, High Fat Diet Slim; Dashed white, Peyer’s patches area.

The colon histomorphology was evaluated by the external muscular layer (Supplementary Figure S3A) and the mucosal layer (Supplementary Figure S3B) which did not differ between the groups. Morphohistology of the large intestine (colon) was characterized using hematoxylin and eosin (H&E) staining to evaluate morphology, the number of goblet cells, Lieberkühn crypts, and the Auerbach plexus, as shown in Figure 2A. Remarkably, Goblet cells (Figure 2B) and Lieberkühn crypts (Figure 2C) number per linear millimeter was higher in HFD_Slim mice when compared to HFD animals, *p* < 0.05 and *p* < 0.01, respectively. The ratio between the number of Goblet cells and Lieberkühn crypts (Figure 2D) did not present significant differences between the groups. We also evaluated the Auerbach plexuses, both in quantity, height, and width per linear mm. We did not observe significant differences in Auerbach plexuses quantity and height between the groups (Figure 2F). However, we observed that the Auerbach plexus width was significantly greater in the HFD_Slim group than in the HFD group (*p* < 0.05; Figure 2G). Scatter plot to show the Auerbach plexus can be seen in Figure 2H. Morphohistological analysis of Peyer’s patches in the large intestine (colon) was also performed using H&E staining, as presented in Figure 3A. We evaluated the Peyer’s patches in terms of quantity, height, and width per linear mm, as shown in Figures 3B–D. Interestingly, Peyer’s patches were significantly larger in the HFD_Slim group in quantity, height, and width, when compared to the HFD group, with *p* < 0.5 in all parameters. Scatter plot to show the Peyer’s patches can be seen in Figure 3E.

### 3.3 Slim nutraceutical recover correlations in gut microbiota after 4 weeks of supplementation

We evaluated the obese mice gut microbiota profile (phylum, genus, species, and *α*-diversity) Pearson correlation with plasma lipid profile and colon histomorphology (goblet cells and Lieberkühn crypts). We observed a significant positively correlation between the VLDL-c and triglycerides and the *Mucispirillum schaedleri*, since phylum (*Deferribacteres*) and Genus (*Mucispirillum*), and *AF12* genus was correlation with plasmatic VLDL-c with the HFD group. However, after 4 weeks of the Slim supplementation this correlation was not observed in Slim_HFD group, like in NFD group.

**Figure 4:**
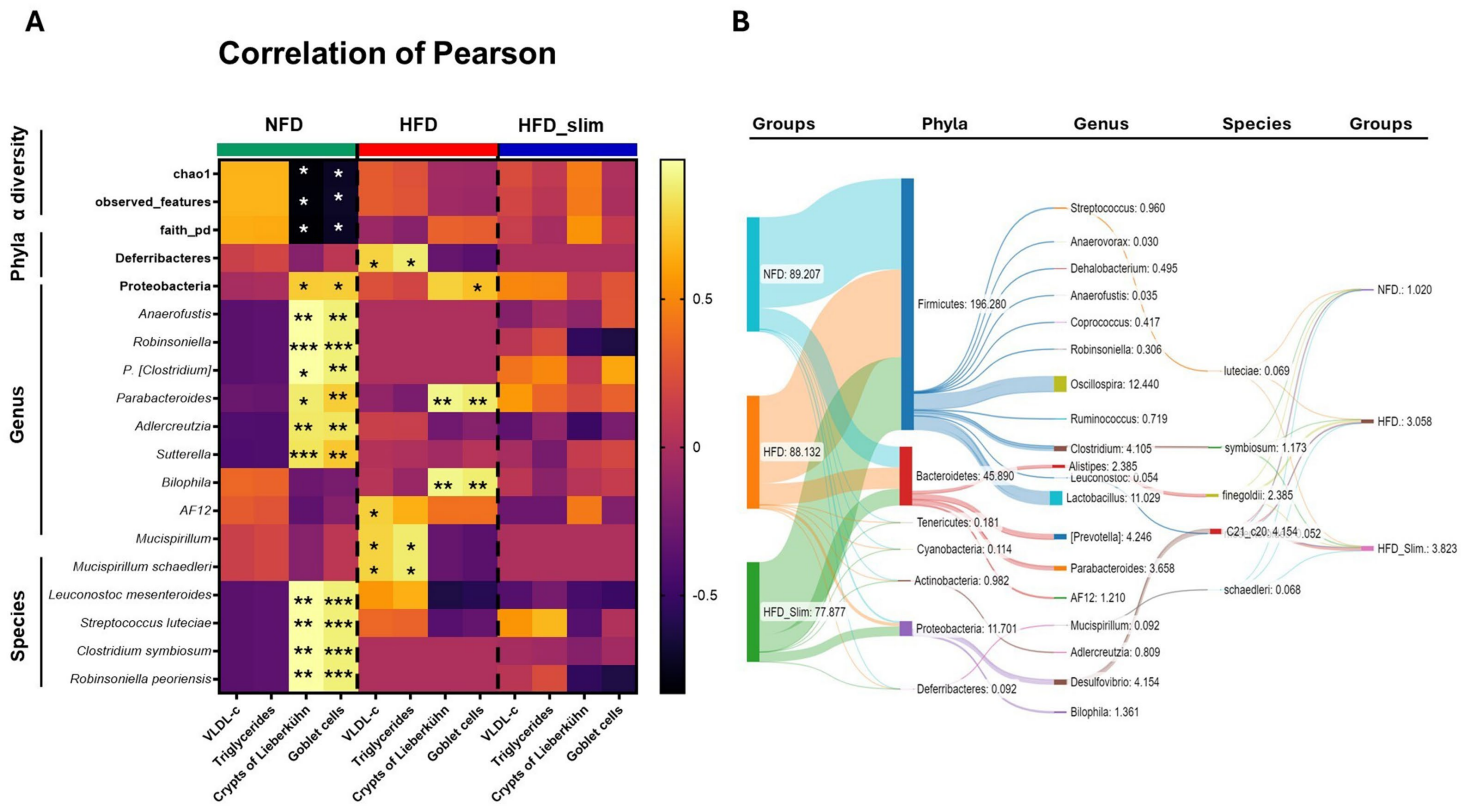
Person correlation analysis between α-diversity, phyla, genera, species, colon histomorphology, VLDL cholesterol and triglycerides of Control and High-Fat Diet groups after 4 weeks of supplementation. **(A)** Heatmap (correlation matrix) Pearson correlation coefficient (R) between the relative abundance (%) of bacteria α-diversity, phyla, genera, species, colon histomorphology, VLDL cholesterol and triglycerides of supplemented mice after 4 weeks. **(B)** Sankey diagram showing the relationship between phylum, genus, and species of bacteria from the intestinal mucosa of NFD, HFD, and HFD_Slim mice. *p < 0.05; **p < 0.01; ***p < 0.001. NFD, Non-Fat Diet; HFD, High Fat Diet; HFD_Sim, High Fat Diet Slim.

Lieberkühn crypts and Goblet cells in the NFD group shown statistical correlated positively concomitantly with the phylum *Proteobacteria* (phylum), the genera *Adlercreutzia, Parabacteroides, Leuconostoc, Anaerofustis, Robinsoniella, P. [Clostridium],* and *Sutterella* (genera), and *Leuconostoc mesenteroides, Streptococcus luteciae, Clostridium symbiosum* and *Robinsoniella peoriensis* (species), and negatively correlated with α-diversity chao1, observed_features, and faith_pd. In the HFD group, we observed a positive correlation between Lieberkühn crypts and Goblet cells and the *Parabacteroides* and *Bilophila* genera. Goblet cells were also positively correlated with *Leuconostoc.* Interestingly, we did not observe significance correlations in the HFD_Slim group (Figure 4A). Sankey diagram showed flow and connections between phylum, genus, and species relative abundance in the gut microbiota groups in the groups (Figure 4B). We can observed the efficiency of Slim supplementation increasing the abundance of species in HFD_Slim group when compared with the other groups (Figure 4B). Main observations in the HFD_Slim group was able to decrease the gut microbiota abundance of Cyanobacteria and Deferribacteres phyla, *Oscillospira*, P. *[Prevotella]*, *Alistipes*, *Bilophila*, *AF12*, *Coprococcus*, *Adlercreutzia*, *Dehalobacterium* and *Mucispirillum* genera; and *Alistipes finegoldii and Mucispirillum schaedleri* species. However, Slim HFD increased Proteobacteria phylum, *Parabacteroides, Sutterella*, *Anaerofustis*, *Robinsoniella*, P. *[Clostridium]*genera, and *Clostridium symbiosum* and *Robinsoniella peoriensis* species when compared to the HFD group (Figure 4B).

### 3.4 Slim nutraceutical modulates alpha and beta diversity in obese mice after 4 weeks of supplementation

**Figure 5:**
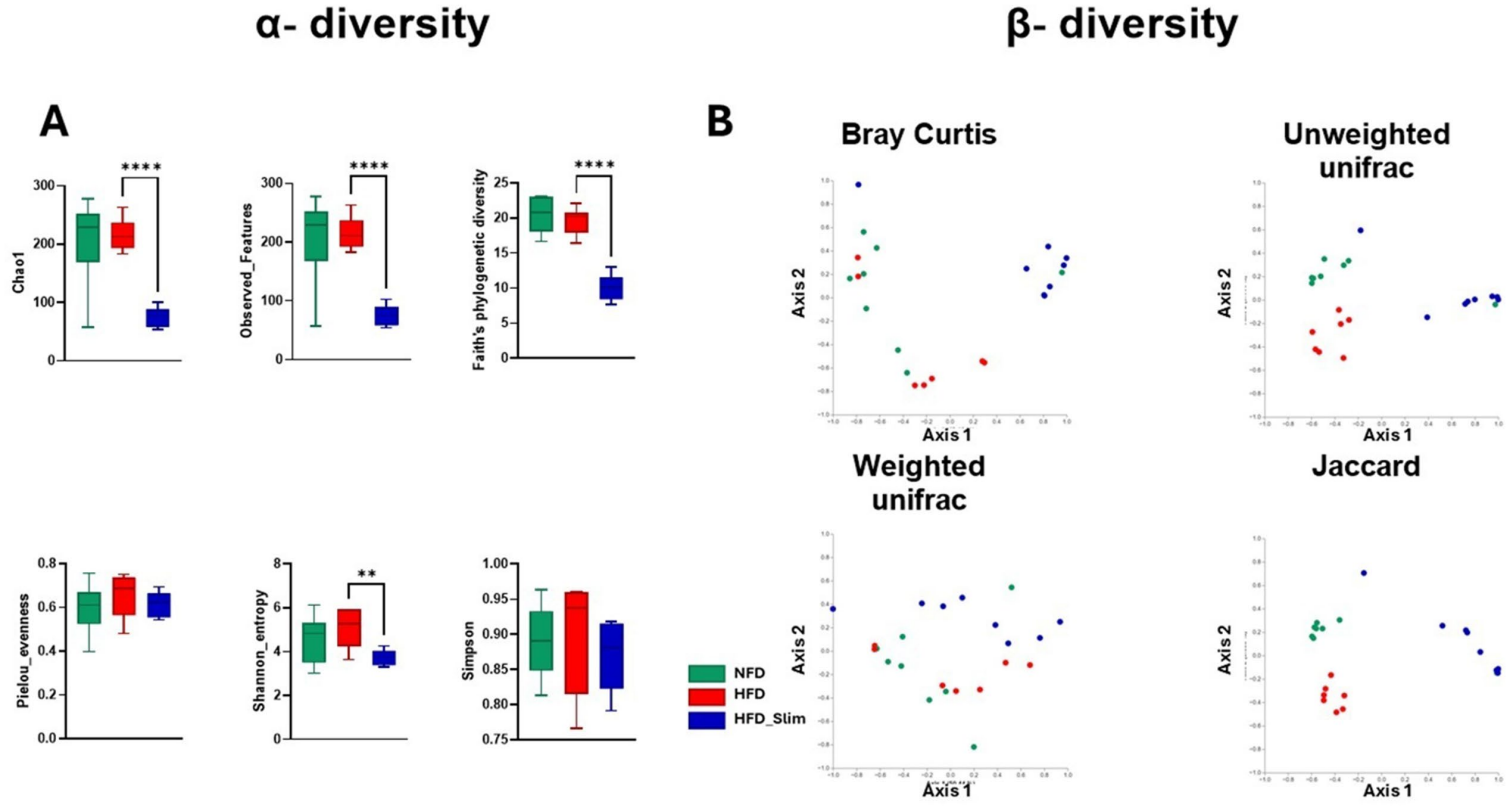
Alpha diversity (α) and beta diversity (β) in the gut microbiota in the Control and High-Fat Diet groups after 4 weeks of supplementation. **(A)** Alpha diversity (α) index. Chao1, Observed_Featured, Faith_pd, Pielou_evenness, Shannon_entropy and Simpson. **(B)** Beta diversity (β) index. Bray-Curtis, Jaccard, Unweighted_unifrac, Weighted_unifrac. Statistical analyses were represented by Student’s t-test or Kruskal-Wallis. Data are represented as mean ± SD. *n* = 7–8 animals per group. Differences were calculated relative to the HFD group. **p < 0.01 and ****p < 0.0001. NFD, Non-Fat Diet; HFD, High Fat Diet; HFD_Slim, High Fat Diet Slim.

We observed significant differences in alpha diversity indexes (α) between the HFD and HFD_Slim groups. Specifically, Chao1 (*p* < 0.0001), Observed_Features (*p* < 0.0001), Faith’s phylogenetic diversity (*p* < 0.0001), and Shannon entropy (*p* < 0.01) were all notably lower in the HFD_Slim group when compared with HFD. The analysis showed no differences between the groups regarding the Pielou Evenness and Simpson parameters (Figure 5A).

*β* diversity comparisons were evaluated using various methods: Bray–Curtis dissimilarity analysis, which considers abundances but not phylogenetic relationships; Unweighted UniFrac and Weighted UniFrac, which incorporate phylogenetic information; and the Jaccard index, which ignores exact abundances and focuses solely on presence/absence values. These analyses were visualized through Principal Coordinate Analysis (PCoA) plots, which indicated whether the samples clustered according to their bacterial composition (Figure 5B). Comparisons of β-diversity indicated significant differences in the composition of the mouse microbiota between the groups evaluated, as shown in Figure 5B. The differences that were found are established at the phylogenetic level. PERMANOVA indicated significant differences in β-diversity [Bray-curtis: HFD versus NFD *p* < 0.046 and HFD versus HFD_Slim (*p* < 0.002); Jaccard: HFD versus NFD *p* < 0.001 and HFD versus HFD_Slim (*p* < 0.001); Weighted_unifrac: HFD versus NFD *p* < 0.272 and HFD versus HFD_Slim (*p* < 0.075); Unweighted_unifrac: HFD versus NFD *p* < 0.001 and HFD versus HFD_Slim (*p* < 0.001) (Figure 5B)]. PERMDISP did not show significant differences in beta diversity [Bray-Curtis: HFD versus NFD *p* < 0.915 and HFD versus HFD_Slim (*p* < 0.796); Jaccard: HFD versus NFD *p* < 0.811 and HFD versus HFD_Slim (*p* < 0.072); Weighted_unifrac: HFD versus NFD *p* < 0.519 and HFD versus HFD_Slim (*p* < 0.462); Unweighted_unifrac: HFD versus NFD *p* < 1.000 and HFD versus HFD_Slim (*p* < 0.732) (Figure 5B)]. ANOSIM did not show significant differences in beta diversity [Bray-Curtis: HFD versus NFD *p* < 0.046 and HFD versus HFD_Slim (*p* < 0.002); Jaccard: HFD versus NFD *p* < 0.001 and HFD versus HFD_Slim (*p* < 0.001); Weighted_unifrac: HFD versus NFD *p* < 0.208 and HFD versus HFD_Slim (*p* < 0.033); Unweighted_unifrac: HFD versus NFD *p* < 0.002 and HFD versus HFD_Slim (*p* < 0.001) (Figure 5B)].

### 3.5 Slim nutraceutical influences gut microbiota in obese mice after 4 weeks of supplementation

**Figure 6:**
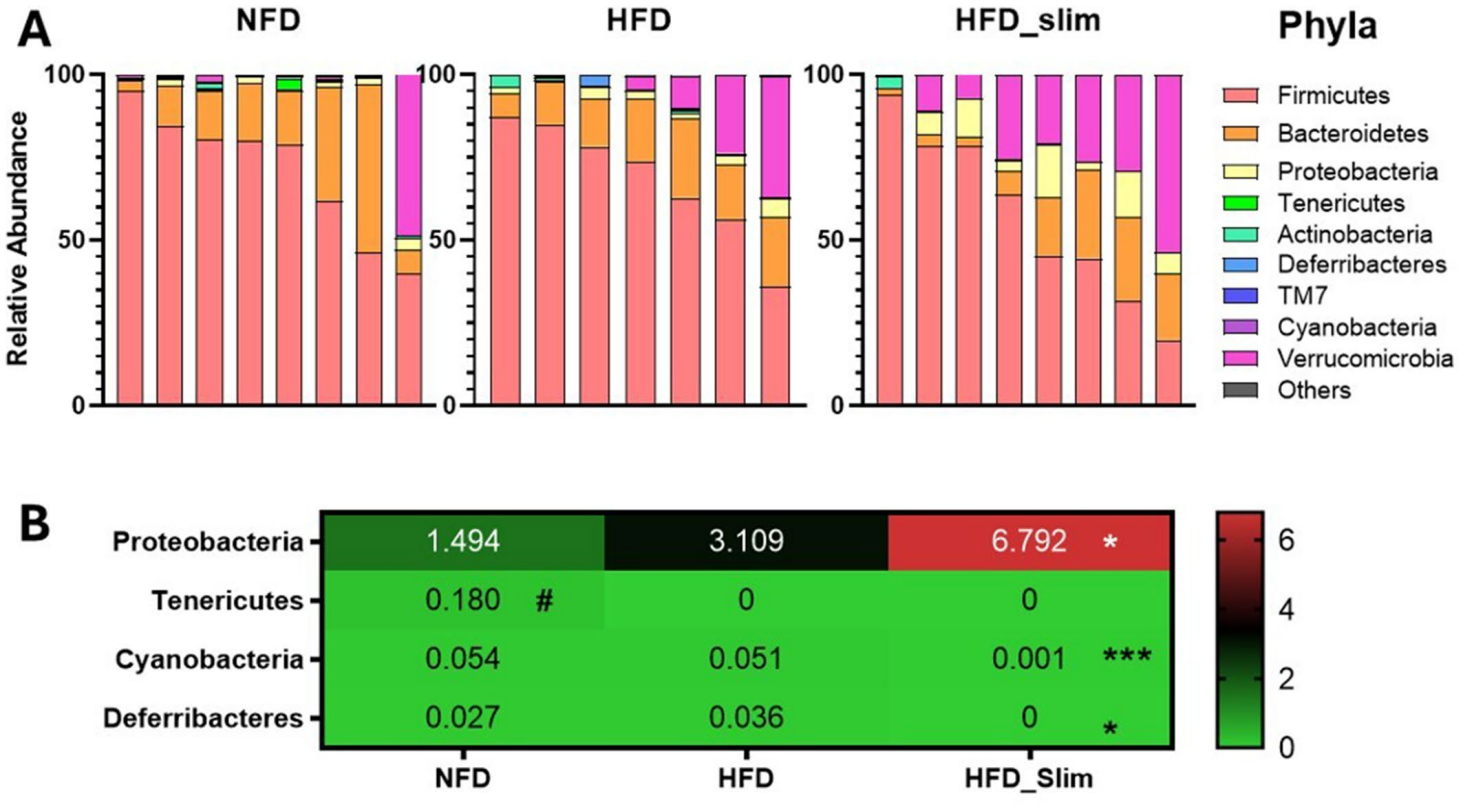
Composition of relative abundance of gut microbiota phyla in the Control and High-Fat Diet groups after 4 weeks of supplementation. **(A)** Relative abundance of phyla. **(B)** Heat map of relative abundance of phyla with statistical differences. Data were represented as mean ± SD. *n* = 7–8 animals per group. (#) Means the difference between NFD and HFD. (*) Means the difference between HFD and HFD_Slim. #*p* < 0.01; *p < 0.0,5, **p < 0.01, and ***p < 0.001. NFD, Non-Fat Diet; HFD, High Fat Diet; HFD_Slim, High Fat Diet Slim.

Relative abundance of the gut microbiota phyla (Figure 6A). After 4 weeks of supplementation, we observed the abundance decrease Cyanobacteria (*p* < 0.001) and Deferribacteres (*p* < 0.05), and increased in the Proteobacteria (*p* < 0.05) in in the gut microbiota composition of the HFD_Slim when compared to the HFD group (Figure 6B). Tenericutes (*p* < 0.05) abundance was increase in NFD group when compared to the HFD group (Figure 6B).

**Figure 7:**
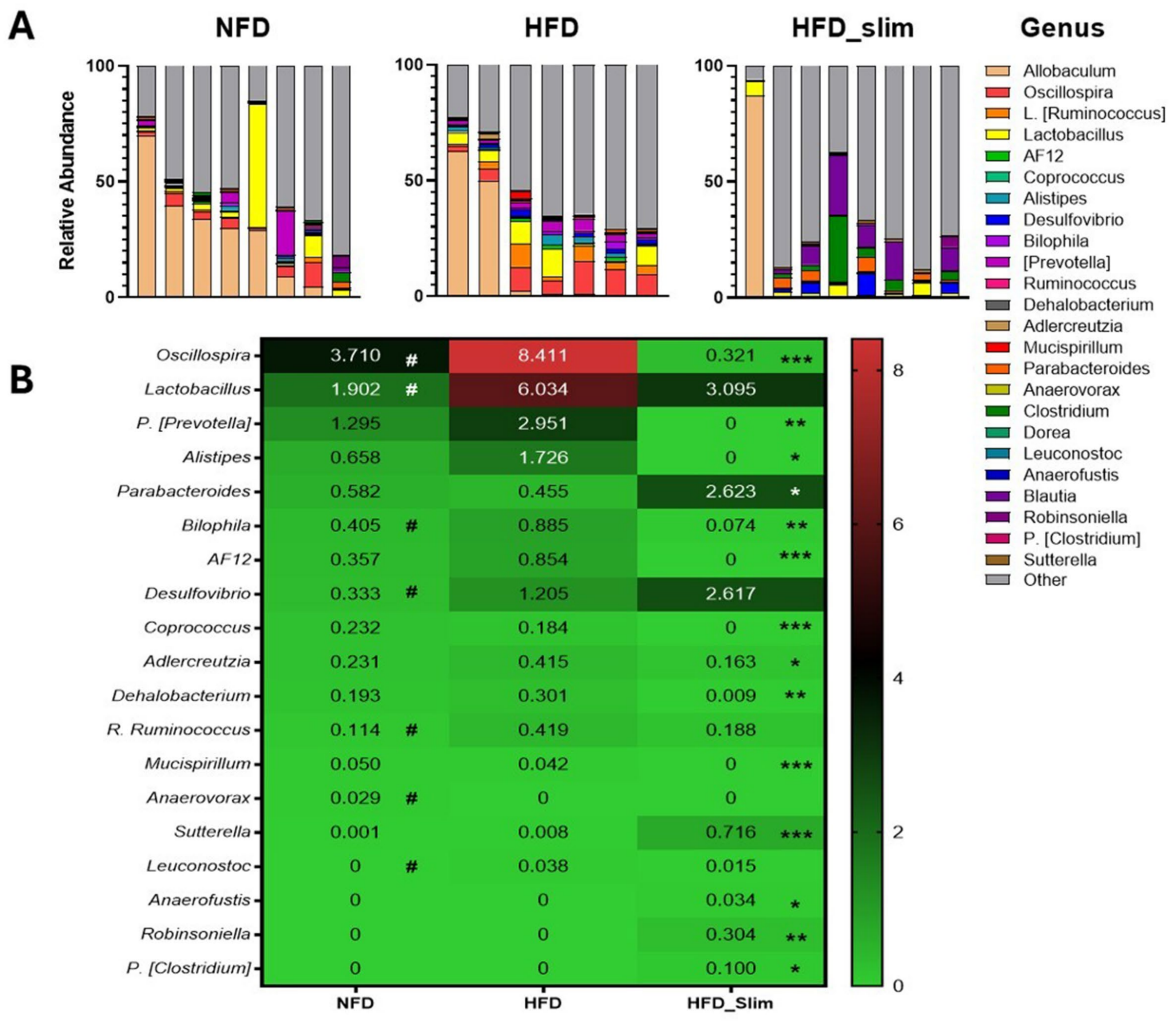
Composition of gut microbiota genera in the Control and High-Fat Diet groups after 4 weeks of supplementation. **(A)** Relative abundance of genera. **(B)** Heat map of relative abundance of genera with statistical differences. Data were represented as mean ± SD. *n* = 7–8 animals per group. (#) Means the difference between NFD and HFD. (*) Means the difference between HFD and HFD_Slim. #*p* < 0.01; *p < 0.0,5, **p < 0.01, and ***p < 0.001. NFD, Non-Fat Diet; HFD, High Fat Diet; HFD_Slim, High Fat Diet Slim; NFD, Non-Fat Diet; HFD, High Fat Diet; HFD_Slim, High Fat Diet Slim.

Relative abundance of the gut microbiota genera (Figure 7A). We observed abundance decrease in *Oscillospira* (*p* < 0.05), *Lactobacillus* (*p* < 0.05), *Bilophila* (*p* < 0.05), *Desulfovibrio* (*p* < 0.05), R. *Ruminococcus* (*p* < 0.05), *Leuconostoc* (*p* < 0.05), and abundance increase in *Anaerovorax* (*p* < 0.05) in NFD group when compared with the HFD group (Figure 7B). After 4 weeks of the Slim supplementation was able to decrease *Oscillospira* (*p* < 0.001), P. *[Prevotella]* (*p* < 0.01), *Alistipes* (*p* < 0.05), *Bilophila* (*p* < 0.01), *AF12* (*p* < 0.001), *Coprococcus* (*p* < 0.001), *Adlercreutzia* (*p* < 0.01), *Dehalobacterium* (*p* < 0.01), *Mucispirillum* (*p* < 0.001) and increase *Parabacteroides* (*p* < 0.05), *Sutterella* (*p* < 0.001), *Anaerofustis* (*p* < 0.05), *Robinsoniella* (*p* < 0.01), P. *[Clostridium]* (*p* < 0.05) gut microbiota abundance when compared with the HFD group (Figure 7B).

**Figure 8:**
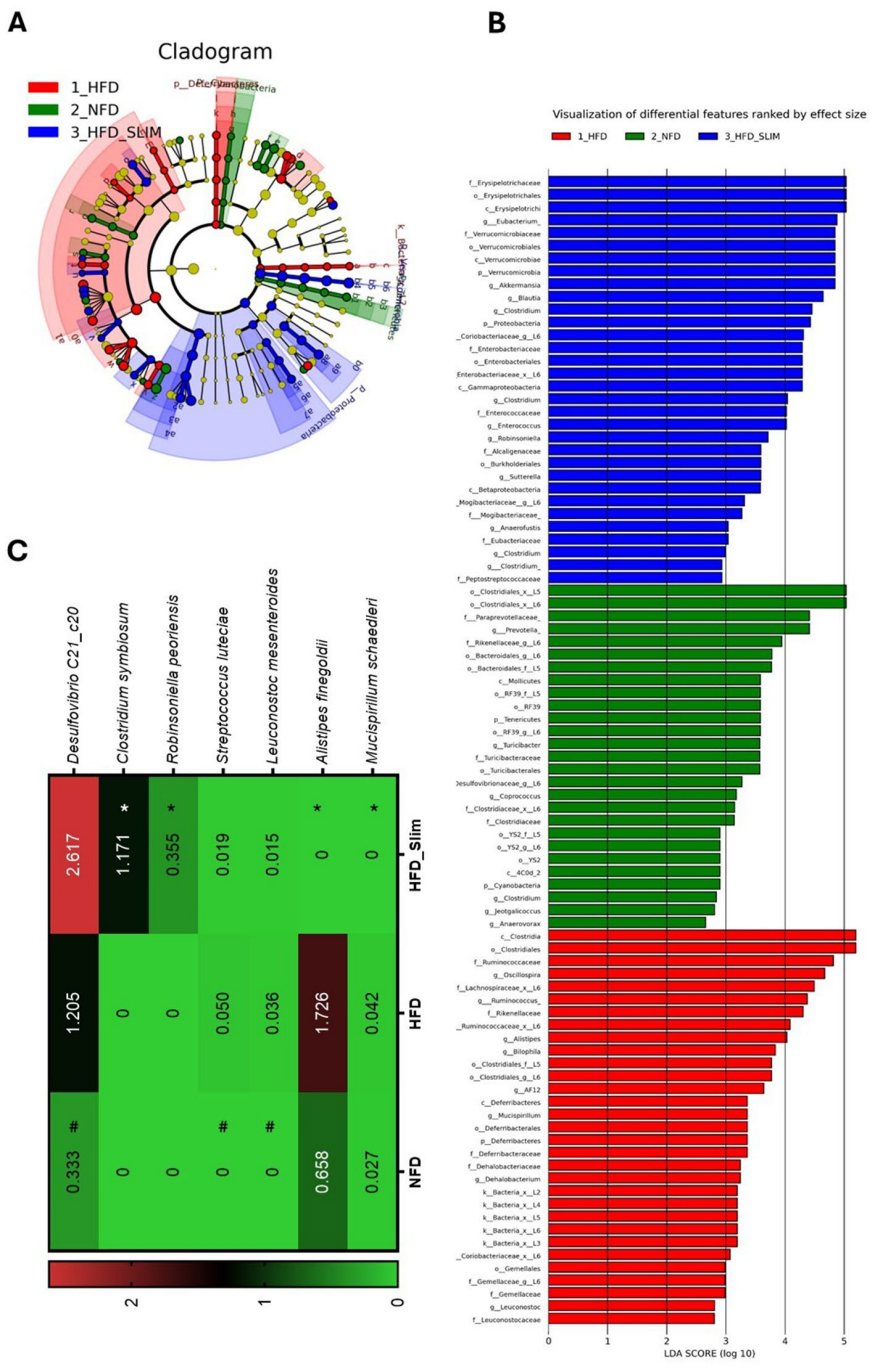
Differentially enriched bacterial taxons by LEfSe in gut microbiota of the Control and High-Fat Diet groups after 4 weeks of supplementation. **(A)** Cladogram of significant changes across taxonomic levels. The root of the cladogram represents the bacteria domain. Node size represents taxon abundance. The most abundant enriched taxon among the NFD (blue), HFD (green), and HFD_Slim (red) groups. **(B)** Graphical representation of the most abundant bacterial feature. Only taxon meeting an LDA significance threshold >2 is shown. **(C)** Heat map of the relative abundance of species with statistical differences. Data were represented as mean ± SD. *n* = 7–8 animals per group. Differences were calculated relative to the HFD group. #*p* < 0.01 – significant difference between NFD and HFD; *p < 0.05, **p < 0.01, and ***p < 0.001 – significant difference between HFD_Slim and HFD. NFD, Non-Fat Diet; HFD, High Fat Diet; HFD_Slim, High Fat Diet Slim.

LEfSe is an algorithm for high-dimensional biomarker discovery that identifies genomic features, such as genes, pathways, or taxa, characterizing differences between multiple biological conditions or classes (Figure 8). LEfSe analysis was used to identify the primary phylotypes responsible for the differences between the groups. This analysis helped to discern significantly and biologically distinct features, considering the importance of these effects and allowing for the identification of differentially abundant features (Segata et al., 2011). Linear discriminant analysis (LDA), combined with effect size measures (LEfSe) and derived cladogram, shows the significantly abundant bacterial taxa belonging to the NFD, HFD, and HFD_Slim groups (Figures 8A,B). The HFD_Slim supplementation was able to rise the *Eubacterium; Akkermansia; Clostridium; Enterococcus; Robinsoniella; Sutterella; Anaerofustis;* and *Clostridium* genera, and Erysipelotrichaceae family. The *Prevotella; Turicibacter; Coprococcus; Jeotgalicoccus; Clostridium; Anaerovorax* genera were observed in the composition of the relative abundance of the microbiota of the NFD group while the *Oscillospira; Ruminococcus; Allosprirae; Bilophila; AF12; Mucispirillum; Dehalobacterium; Leuconostoc* genera were observed in the composition of the relative abundance of the microbiota of the HFD group (Figure 8B). The HFD_Slim group showed *Mucispirillum schaedleri* (*p* < 0.05), *Alistipes finegoldii* (*p* < 0.05) abundance decreased, and *Clostridium symbiosum* (*p* < 0.05), and *Robinsoniella peoriensis* (*p* < 0.05) abundance decreased when compared to the HFD group (Figure 8C). *Leuconostoc mesenteroides* (*p* < 0.05), *Streptococcus luteciae* (*p* < 0.05) and *Desulfovibrio C21_c20* (*p* < 0.05) genera showed abundance decreased in NFD group compared to HFD group (Figure 8C).

## 4 Discussion

Bioactive compounds have shown potential as effective interventions to restore intestinal microbiota balance in obesity-related dysbiosis. The growing trend of using plant- and food-derived nutraceuticals as a complementary approach to treating health conditions reflects a shift toward more natural solutions (Rajapakse and Gantenbein, 2024). Our recent studies, both preclinical and clinical, have shown promising results in improving parameters related to obesity and overweight, especially through modulations in the intestinal microbiota with the use of nutraceutical compositions containing prebiotics, minerals, β-glucans and silymarin (Nehmi et al., 2021; Santamarina et al., 2022, 2024; Nehmi-Filho et al., 2023a, 2023b). In order to propose a nutraceutical formulation with a new diversity of active ingredients, we evaluated an innovative formulation named Slim, containing beet pulp, coenzyme Q10, berberine, prebiotics (fructooligosaccharides and galactooligosaccharides), minerals (selenium and chromium), and yeast β-glucans (from *Saccharomyces cerevisiae*), on the lipid profile, colon histomorphology shape, and in the gut microbiota in a high-fat diet obese mice.

The Slim supplement was formulated based on its components’ functions and their synergistic effects. Berberine, a plant alkaloid from *Berberis vulgaris* Rupr., is recognized for a bioactive improvement metabolic syndrome like insulin sensitive, and anti-inflammatory effect on gut microbiota (Cheng et al., 2009; Habtemariam, 2020; Zhang et al., 2021). Beet pulp powder, rich in fiber and antioxidant compounds, like betaine, not only promotes intestinal health but also exerts prebiotic effects, encouraging the growth of beneficial bacteria (Prandi et al., 2018; Adekolurejo et al., 2023). Coenzyme Q10, a powerful antioxidant, protects against oxidative stress and improves mitochondrial function, crucial factors for metabolic.

One of our key objectives was to propose a formulation to achieve weight loss or reduce weight gain in obese mice model. While we observed this outcome in our previous studies (Nehmi et al., 2021), we did not see the same effect with the Slim supplement (see Supplementary Figure 1). However, we did note a reduction in plasma levels of VLDL cholesterol, triglycerides, and HDL cholesterol (Table 1), which supports our preclinical findings in the same high-fat diet-induced obesity model (see our first and second papers).

In our investigation of the Slim nutraceutical effects on lipid metabolism, we decided to assess the histomorphology of the colon. This choice is based on the understanding that prebiotic nutraceuticals influence not only the intestinal microbiota but also the host microenvironment (Santamarina et al., 2022). It is known that large intestine (colon) histomorphology and microbiota of the mice fed a high-fat diet significantly differ when compared with mice fed with normal-calorie diet (Paresque et al., 2013; John and Mullin, 2016; Nehmi-Filho et al., 2023a).

Goblet cells play a vital role in the production and secretion of mucus in the intestine. This mucus is crucial for maintaining the integrity of the intestinal barrier (Yang and Yu, 2021). It forms a hydrophobic barrier in the intestinal mucosa that reduces fat absorption (Lichtenberger, 1995) while enhancing nutrient uptake and supporting metabolic homeostasis. Our results showed that obese mice receiving Slim supplementation exhibited a significant increase in both Goblet cells and Lieberkühn crypts compared to those on a high-fat diet (HFD) (Figures 2B,C). This increase may explain the observed reduction in plasma VLDL-c and triglycerides in Slim HFD (Table 1).

We observed a positive Pearson correlation between the Parabacteroides and Bilophila genera with Goblet cells and Lieberkühn crypts in the high-fat diet (HFD) group (Figure 4A). Parabacteroides is associated with the fermentation of complex polysaccharides and producing short-chain fatty acids (SCFAs), such as propionate (Medawar et al., 2021). *Bilophila* spp., a sulfate-reducing bacteria (SRB) seems to reduce butyrate-producing bacteria level (Ye et al., 2018). We observed a decreased and increased abundance in these genera, respectively, in the HFD group and an inverse result in the NFD and Slim HFD group after 4 weeks (Figure 4A). *Mucispirillum schaedleri*, a marker of a high-fat diet, showed a positive Pearson correlation with VLDL cholesterol and triglyceride levels, even as in the *Mucispirillum* genus in the HFD group, but we did not observe this phenotype in the NFD and HFD_Slim groups (Figure 4A). These results suggest a recovery of histomorphology and modulation of gut microbiota, supporting the known interrelation between the gut microbiota and the host microenvironment.

Obesity and high-fat diets can negatively impact gut-associated lymphoid tissue (GALT), including Peyer’s patches (Jung et al., 2010; Leocádio et al., 2020). These factors contribute to chronic inflammation, reduced motility, and dysfunction of the enteric nervous system (ENS) (McMenamin et al., 2018; Miron, 2019), which may result in constipation and bacterial overgrowth. Peyer’s patches are crucial for mucosal immune responses, while Auerbach’s plexus is a network within the ENS that is essential for intestinal motility (Sharkey and Mawe, 2023). After 4 weeks of Slim supplementation, both Peyer’s patches (Figure 3B) and Auerbach’s plexus (Figure 2E) showed significant expansion. One noteworthy observation was that the anti-inflammatory effects of the components in the Slim formulation did not appear to impact the intestinal microenvironment, including histomorphology and microbiota. Although the Slim formulation contains anti-inflammatory characteristics, the analysis of the cytokines IL-6 and IL-10 in the colon of the treated groups showed no significant changes due to the supplement (Supplementary Figures S2D–F).

Lower alpha diversity is often associated with intestinal dysbiosis (Sharma et al., 2023), which may contribute to the development and persistence of obesity. However, in certain contexts, a reduction in alpha diversity can indicate a healthier state and a lower risk of complications and infections (The Human Microbiome Project Consortium, 2012; Baud et al., 2023). On the other hand, some studies suggest that increased alpha diversity might be linked to adverse outcomes (Kupritz et al., 2021). In contrast, beta diversity plays a critical role in understanding regional diversity, as it measures the similarity or dissimilarity between pairs of microbiomes (Su, 2021). We obtained some intriguing results with the Slim supplement (Figure 5B), which contradicted our earlier studies that utilized a different type of polyphenol (Nehmi-Filho et al., 2023a; Nehmi-Filho et al., 2024). In the HFD_Slim group, we observed a decrease in alpha diversity (diversity within each group) and an increase in beta diversity (diversity between groups) (Figures 5A,B). This indicates that the nutraceutical was able to modulate diversity when compared to the different groups evaluated.

Proteobacteria phylum is thought to play a key role in preparing the gut for intestinal mucosalization by strictly anaerobic bacteria necessary for healthy gut function by consuming oxygen and reducing the redox potential in the gut environment (Moon et al., 2018). The Proteobacteria abundance augmented (Figure 4A) indicates that the positive effects observed on gut diversity, histomorphology, and lipid metabolism, along with the reduction of the phyla Cyanobacteria and Deferribacteres after Slim supplementation, may be linked to the potential duplibiotic effect from the supplement (Figure 4A). This effect is believed to arise from the polyphenols in Beet pulp and Berberine (Habtemariam, 2020), which may exert an antimicrobial action to control and inhibit the growth of pathogenic bacteria while also having a prebiotic effect that stimulates the growth of beneficial, commensal bacteria (Wang et al., 2023; Rodríguez-Daza et al., 2021). Furthermore, polyphenols can alleviate metabolic diseases, increase mucus production, and promote the secretion of antimicrobial peptides (Rodríguez-Daza et al., 2021). Although the duplibiotic effect should not be mitigated, we believe the results obtained were due to the combination of active ingredients rather than any single isolated compound. This conclusion is supported by the fact that the suggested quantities of components, such as FOS and GOS, were below those recommended in the literature (Sabater-Molina et al., 2009; Tonon et al., 2021).

The Slim supplement shows its prebiotic effect in increasing the abundance of important genera that bring benefits in improving conditions associated with the obesity-induced model (Figure 7A). *Desulfovibrio* is a potent generator of acetic acid, which showed significant anti-NAFLD (Non-alcoholic fatty liver disease) effects in HFD-fed mice (Hong et al., 2021). *Sutterela* shows the ability to adhere to intestinal epithelial cells, indicating that they may have an immunomodulatory role (Hiippala et al., 2016). *Parabacteroides* is associated with the fermentation of complex polysaccharides and producing short-chain fatty acids (SCFAs), such as propionate (Medawar et al., 2021; Figure 7B). *Akkermancy,* a genus that plays a role in the renewal and production of mucin, can also influence the differentiation of Paneth and Goblet cells in the small intestine (Schneeberger et al., 2015; Kim et al., 2021). In our experiment, we observed an increase in these cell types in the colon, suggesting that *Akkermancy* may modulate their development (Figure 8B). The antibiotic effect of the Slim formulation was evident in the reduction of the genera *Bilophila* (Ye et al., 2018) and *Mucispirillum* (Herp et al., 2021). These genera are responsible for lowering the levels of butyrate-producing bacteria (BPB) and for degrading mucin, respectively (Figure 7B). The Slim supplement was able to influence some bacterium species that bring benefits in obese mice model increasing *Desulfovibrio C21-C20*, *Clostridium symbiosum* (Luo et al., 2023), and reducing *Mucispirillum schaedleri* (Loy et al., 2017), *Alistipes finegoldii* (Parker et al., 2020; Figure 8C) that seems involved in homeostasis breakdown in obese-gut microbiota.

## 5 Conclusion

In conclusion, the findings of our study support the potential of Slim as a nutraceutical intervention for obesity-related dysbiosis, enhancing gut health and mitigating some of the metabolic disruptions caused by high-fat diets. Although our findings are promising, we acknowledge several limitations in this study. First, it was not possible to assess the individual effects of beet pulp, coenzyme Q10, and berberine. Second, we were unable to measure short-chain fatty acids in the fecal samples of the experimental groups. While our sample size was adequate, a larger sample size would enhance the robustness of the study and increase confidence in the reproducibility of our results. Additionally, a more in-depth evaluation of the intestinal barrier is needed to better understand its function. Future studies in both animal and human models will be essential to address these important questions.

## Data Availability

The datasets presented in this study can be found in online repositories. The names of the repository/repositories and accession number(s) can be found at: https://www.ncbi.nlm.nih.gov/genbank/, PRJNA941000.
