# Supplementary material for: Nutraceutical supplement slim reshaped colon histomorphology and reduces Mucispirillum schaedleri in obese mice
Source: Front Microbiol. 2025 Apr 1;16:1494994. doi: 10.3389/fmicb.2025.1494994 (PMC11997693; doi:10.3389/fmicb.2025.1494994)

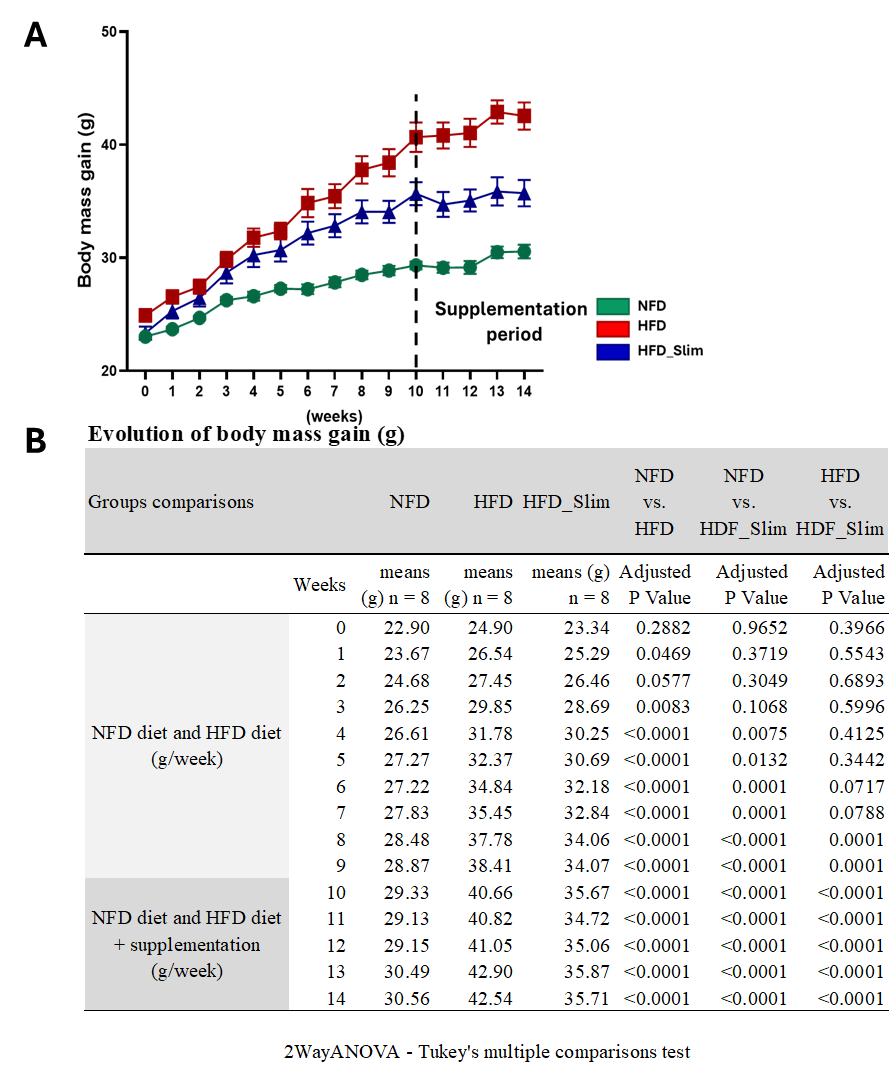


**Figure 1S. Evolution of body mass gain (grams).** (A) Evolution of weight gain in mice throughout the development of obesity and supplementation. Means and statistical analyses are shown in supplementary table 1. (B) Table with descriptive data of evolution of weight gain. Statistical analyses were represented by 2WayANOVA - Tukey's multiple comparisons test. Data were represented as mean, n = 8 animals per group. * p<0.05; ** p<0.01; *** p<0.001; **** p<0.0001.

NFD= Non-Fat Diet; HFD= High Fat Diet; HFD_Slim= High Fat Diet slim.

**
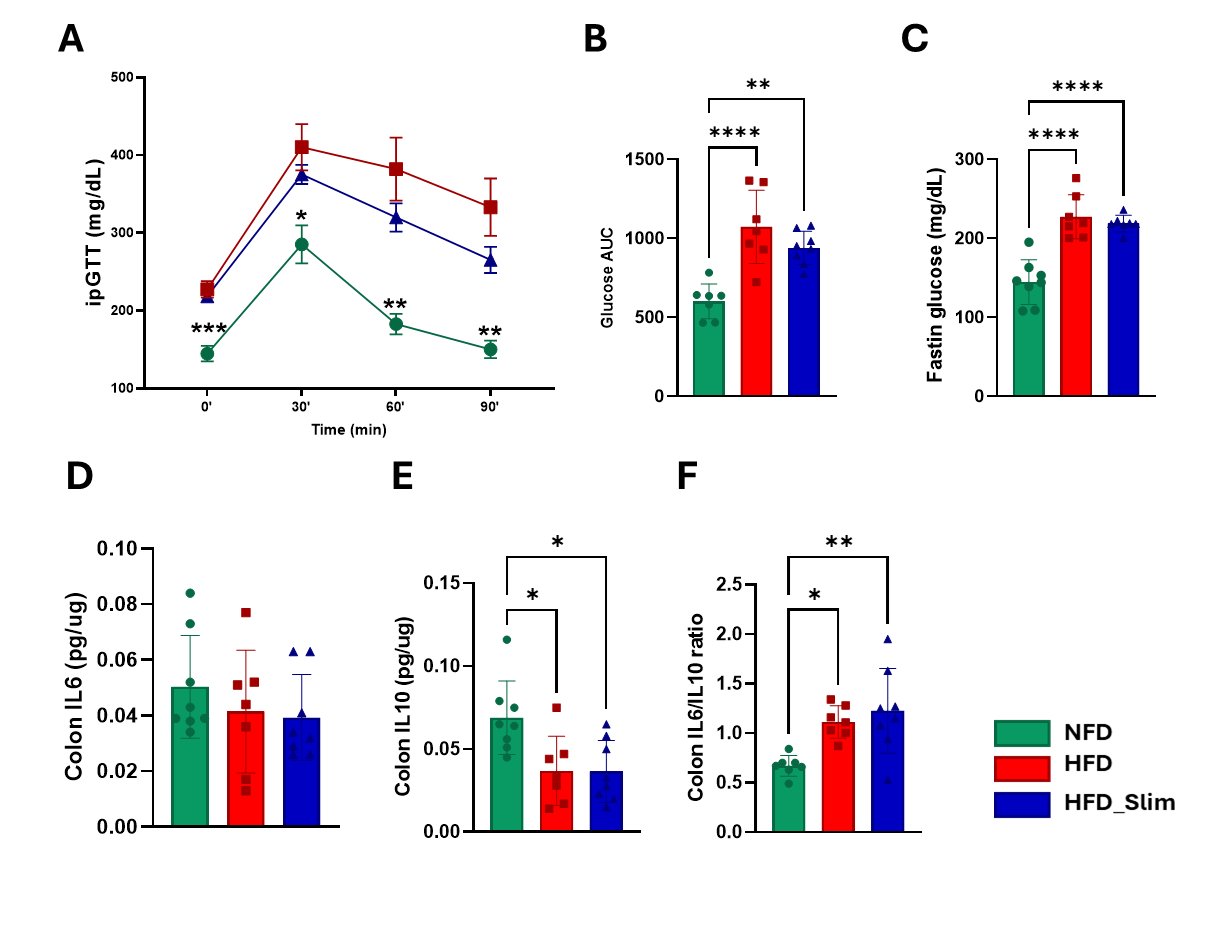
**

**Figure 2S. Glycemic parameters and cytokines in the colon of NFD, HFD and HFD_Slim groups four weeks of supplementation.** (A) intraperitoneal glucose tolerance test (ipGTT); (B) glucose AUC; (C) fasting glucose; (D) Colon Interleukin-6 (IL6); (E) Colon Interleukin-10 (IL10); (F) Colon Interleukin IL6/IL10 ratio. Statistical analyses were represented by Student's t test or Kruskal-Wallis. Data were represented as mean±SD and median and interquartile range. n= 7-8 animals per group. Differences were calculated in relation to the HFD group. * p<0.05; ** p<0.01; **** p<0.0001. NFD= Non-Fat Diet; HFD= High Fat Diet; HFD_Slim= High Fat Diet slim; ipGTT= intraperitoneal glucose tolerance test; AUC= area under the curve.

**Figure 3S. Colon morphological evaluation of mice after 4-weeks supplementation.** (A) muscularis externa; (B) mucosa. Statistical analyses were performed using the student’s t-test or Kruskal-Wallis test. Data are represented as mean±SD. n=7-8 animals per group. Differences were calculated about the HFD group. NFD= Non-Fat Diet; HFD= High Fat Diet; HFD_Slim= High Fat Diet Slim
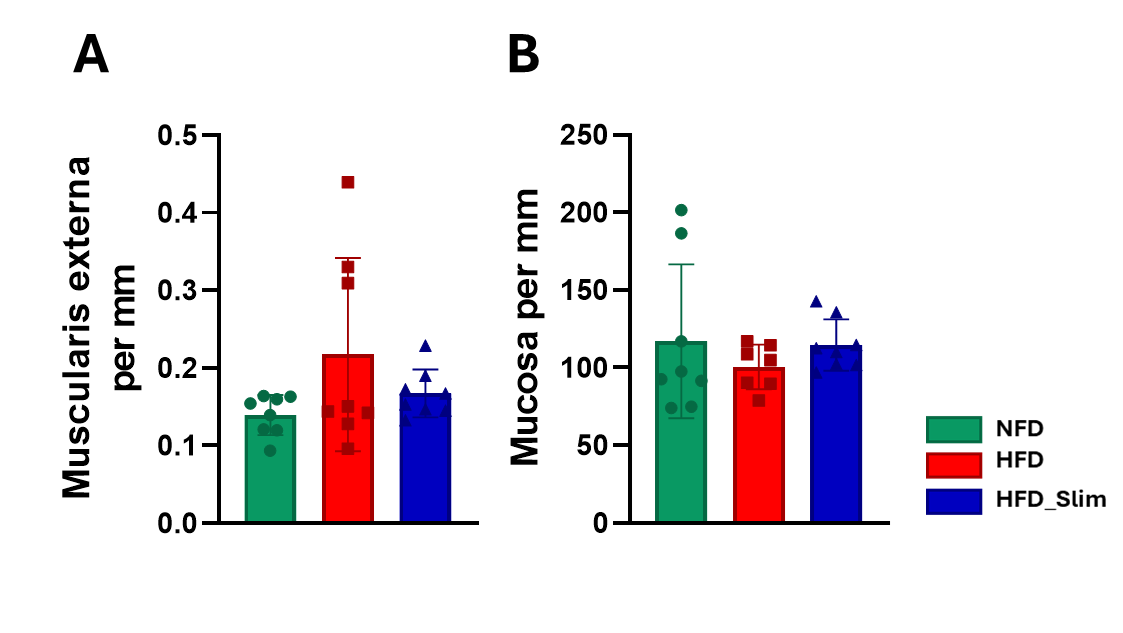

Supplement: Supplementary file 1 [file Data_Sheet_1.docx]
